# Direct Observation of Substantial Phonon Nonequilibrium Near Nanoscale Hotspots in Gallium Nitride

**DOI:** 10.1002/advs.202411040

**Published:** 2025-01-24

**Authors:** Jiaxuan Xu, Xiaona Huang, Yufei Sheng, Qiangsheng Sun, Hongkai Zhang, Hua Bao, Yanan Yue

**Affiliations:** ^1^ School of Power and Mechanical Engineering Wuhan University Wuhan Hubei 430072 P. R. China; ^2^ University of Michigan‐Shanghai Jiao Tong University Joint Institute Shanghai Jiao Tong University Shanghai 200240 P. R. China; ^3^ Global Institute of Future Technology Shanghai Jiao Tong University Shanghai 200240 P. R. China; ^4^ Department of Mechanical and Manufacturing Engineering Miami University Oxford OH 45056 USA

**Keywords:** first‐principles calculations, nanoscale phonon nonequilibrium, phonon Boltzmann transport theory, tip‐enhanced Raman measurement

## Abstract

Phonon modal nonequilibrium is believed to widely exist around nanoscale hotspots, which can significantly affect the performance of nano‐electronic and optoelectronic devices. However, such a phenomenon has not been explicitly observed in 3D device semiconductors at the nanoscale. Here, by employing a tip‐enhanced Raman thermal measurement approach, substantial phonon nonequilibrium in gallium nitride near sub‐10 nm laser‐excited hotspots is directly revealed for the first time. As further evidence, quantitative agreements between measurements and accurate first‐principles‐based phonon Boltzmann transport equation calculations are obtained. The large nonequilibrium is attributed to the strong Fröhlich coupling of electrons with longitudinal optical phonons and the large acoustic‐optical phonon frequency gap in gallium nitride, which is further demonstrated in other common III‐V semiconductors. This work establishes a viable approach for understanding nanoscale nonequilibrium phonon transport and can potentially benefit the future modulation of hot carrier dynamics.

## Graphical abstract

This study achieves direct characterization of intricate nanoscale phonon transport in semiconductors by developing refined a tip‐enhanced Raman thermal measurement technique. For the first time, substantial phonon nonequilibrium is directly observed near sub‐10nm laser‐excited hotspots in gallium nitride, as also evidenced by quantitative agreement with first‐principles‐based phonon Boltzmann transport equation calculations, which potentially benefit future modulation of energy carrier dynamics.

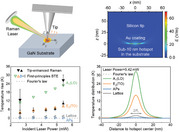

## 1 Introduction

Advancements in nanoscale devices, featuring characteristic dimensions from sub‐micron to even below ten nanometers, are fostering innovations in the fields of electronics, optoelectronics, and photonics.^[^ ^1^, ^2^, ^3^, ^4^ ^]^ The performance of functional devices is intimately coupled to the hot charge carrier transport under photoexcitation or electric bias.^[^ ^5^, ^6^ ^]^ The hot carrier transport in semiconductors is governed by their scattering with phonons to relax excess energy,^[^ ^7^, ^8^ ^]^ and also the subsequent energy dissipation dominated by phonon transport.^[^ ^9^ ^]^ It has been demonstrated that different phonons can be locally driven to deviate from thermal equilibrium under nanoscale optical or electrical excitations due to inadequate phonon thermalization.^[^ ^10^, ^11^, ^12^ ^]^ This manifests as disparate equivalent local temperatures for different phonons.^[^ ^13^, ^14^, ^15^ ^]^ Such phonon (temperature) nonequilibrium has been proven to impede energy dissipation and degrade the thermal reliability of electronic devices,^[^ ^16^, ^17^ ^]^ especially for downscaled fin field‐effect transistors and power electronics.^[^ ^18^ ^]^ In addition, it can also potentially promote slow energy relaxation and even non‐dissipative transport of hot carriers,^[^ ^19^, ^20^ ^]^ which is desired for a variety of promising applications, such as efficient energy‐harvesting optoelectronics,^[^ ^21^ ^]^ thermoelectric cooling devices,^[^ ^7^ ^]^ and broadband photodetectors.^[^ ^3^ ^]^ Therefore, a fundamental understanding of nanoscale phonon nonequilibrium is imperative in the development of post‐Moore‐era nanoelectronics.

While most theoretical studies were based on the multitemperature model,^[^ ^14^, ^22^ ^]^ the phonon Boltzmann transport equation (BTE),^[^ ^23^, ^24^, ^25^, ^26^ ^]^ which can better elucidate the ballistic transport effect, has recently been applied to elucidate phonon nonequilibrium near nanoscale hotspots.^[^ ^18^ ^]^ From the experimental perspective, Raman spectroscopy provides direct measurements of this phonon temperature nonequilibrium according to the Raman spectrum‐temperature dependence.^[^ ^27^, ^28^ ^]^ Experiments based on Raman spectroscopy have illustrated that the weak coupling between in‐plane and out‐of‐plane phonons in graphene^[^ ^29^, ^30^ ^]^ and 2D transition metal dichalcogenides^[^ ^28^, ^31^ ^]^ can easily induce clear phonon nonequilibrium even near microscale laser‐heated hotspots.^[^ ^28^, ^32^ ^]^ However, phonon nonequilibrium has never been explicitly demonstrated in realistic 3D device semiconductors. This is presumably because exciting nanoscale hotspots to facilitate observable phonon nonequilibrium and characterizing the underlying phonon dynamics are quite challenging, particularly for hotspots under tens of nanometers that are extensively generated in modern nanoelectronics.^[^ ^9^ ^]^ Hence, a gap remains in experimental evidence and comprehensive understandings of phonon nonequilibrium in 2D electronic and optoelectronic materials.

In this study, a tip‐enhanced Raman thermal measurement system is developed, which facilitates confined hotspot excitation under the tip apex and achieves sub‐10 nm spatial resolution for characterizing nanoscale phonon temperature rises. We first focus on the wurtzite gallium nitride (GaN), which is extensively favored in optoelectronics^[^ ^33^ ^]^ and high‐power electronics.^[^ ^34^ ^]^ Substantial temperature nonequilibrium among different phonons is directly observed near the excited nanoscale hotspots. A state‐of‐the‐art theoretical framework for phonon BTE integrated with first‐principles calculations is also employed to accurately determine phonon temperatures under nanoscale laser excitations.^[^ ^35^, ^36^ ^]^ Quantitative agreement between Raman measurements and theoretical calculations further evidences this pronounced phonon nonequilibrium induced in GaN. Mechanisms leading to the phonon nonequilibrium in GaN are elucidated from first‐principles‐based phonon BTE results, which are also demonstrated in other common III‐V semiconductors.

## 2 Results and Discussion

### 2.1 Direct Observation of Phonon Nonequilibrium Near Nanoscale Hotspots

**Figure 1:**
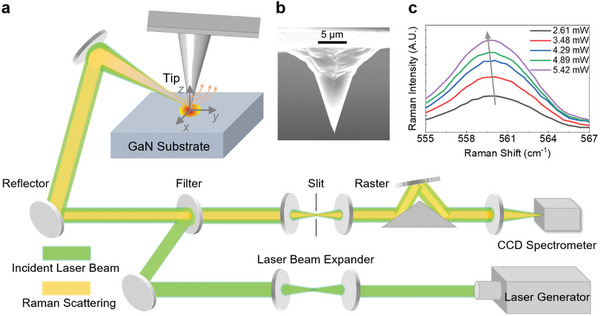
Tip‐enhanced Raman measurement system. a) Schematic of the experimental setup for tip‐enhanced Raman thermal measurement. The incident laser beam is focused at the gold‐coated nanotip apex to heat the GaN substrate and excite Raman signals. The scattered Raman signal is collected by a CCD for temperature probing. b) The SEM image of the gold‐coated silicon nanotip with a half‐cone angle of 20°. c) Raman spectra (*E*_2_(TO) mode) of GaN under different laser excitation (see Supporting Information for details).

**Figure 2:**
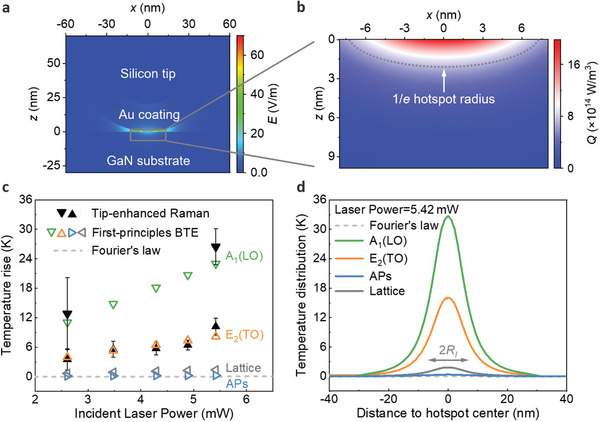
Comparison between tip‐enhanced Raman measurements and first‐principles‐based phonon BTE calculations of the nanoscale hotspot‐induced phonon nonequilibrium. a) Cross‐section view of the electric field distribution *E* around the tip‐substrate contact region (*y* = 0 plane). The coordinate origin is set at the center of the excited hotspot on the surface of the GaN substrate in contact with the nanotip as shown in Figure 1a. b) Heat generation rate distribution *Q* in the GaN substrate induced by the gold‐coated silicon nanotip at a laser power of 5.42 mW. The gray dashed line shows the 1/*e* hotspot radius (i.e., the position at which the peak heat generation rate of the hotspot center drops to 1/*e*). c) Comparison of tip‐enhanced Raman system measured temperature rises for the *E*_2_(TO) phonon mode (upper triangle black‐filled symbols) and *A*_1_(LO) phonon mode (lower triangle black‐filled symbols) and the first‐principles‐based phonon BTE results (hollow symbols) as a function of incident laser power. The dashed line shows temperature rises predicted by macroscale Fourier's law. d) The first‐principles‐based phonon BTE calculated phonon temperature distributions near the excited hotspot at the surface of the GaN sample in contact with the nanotip (*z* = 0 plane) for laser power = 5.42 mW. The gray arrow represents the 1/*e* lateral radius of the hotspot *R_l_*, which is 7.2 nm.

As a wide‐bandgap semiconductor,^[^ ^37^, ^38^ ^]^ GaN has an unusually high lattice thermal conductivity and broadly spanned phonon mean free path distribution (from several nanometers to tens of micrometers).^[^ ^39^ ^]^ These features can potentially lead to clear phonon nonequilibrium in GaN at the nanoscale,^[^ ^16^, ^18^ ^]^ when the characteristic length is smaller than the phonon mean free path. Here, a tip‐enhanced Raman experimental system is employed to conduct the thermal measurement.^[^ ^39^, ^40^ ^]^ The system is customized to feature nanoscale heating from the tip enhancement effect and temperature measurement simultaneously from the Raman signal. As illustrated in **Figure** **1**a, a single crystal GaN is placed on an atomic force microscopy (AFM) sample stage. A gold‐coated silicon nanotip is employed instead of conventional tips to achieve the tip enhancement effect. The system is adjusted as a contact mode between the nanotip and GaN to excite an enhanced optical field.^[^ ^39^ ^]^ Figure 1b displays a SEM image of the silicon nanotip, showing the probe structure used in the measurement. A Raman laser is precisely controlled to focus on the tip apex. Consequently, a significant optical field at the tip‐substrate contact area is formed and such an intense optical field can generate a localized heating region at the subsurface beneath the nanotip. In this configuration, two active modes of GaN are observed, i.e., *E*_2_(TO) and *A*_1_(LO) peaks, which represent the transverse and longitudinal optical phonon modes at the center of the Brillouin zone.^[^ ^41^ ^]^ The average phonon temperature rise of the heated region can be determined from the Raman peak shift (see the Experimental Section for details). The Raman spectra for the *E*_2_(TO) phonon mode at different laser powers are illustrated in Figure 1c. We observe an apparent temperature rise of the *E*_2_(TO) phonon mode from 3.5 to 10.2 K, and the *A*_1_(LO) phonon mode from 12.8 to 26.5 K as the laser power increases from 2.61 to 5.42 mW as shown in **Figure** **2**c (see Supporting Information for details). The strong fields from SERS may cause slight deviations in Raman shifts due to nonlinear effect; however, such an effect is minimized in our work by employing low laser power intensities. Notably, the *A*_1_(LO) mode exhibits relatively weak intensity, necessitating extended accumulation time to generate a sound signal. Thus, only a limited number of signals are recorded (see Supporting Information for details). The small incident angle of the laser (less than 15°) relative to the substrate surface, combined with the low absorptivity of GaN (532 nm in laser wavelength), renders the direct laser heating of the substrate negligible. To verify this, we also conduct an experiment on the GaN substrate without the nanotip. The observed temperature rises, inferred from the Raman shift of the phonon modes, are less than 1 K, significantly lower than the temperature characterized in the presence of the nanotip. In addition, it should be noted that in our experiments, the coated tip is not subjected to significant heating and no substantial thermal stress is induced due to the high reflectivity, shallow skin depth, and high thermal conductivity of the gold coating.

To translate experimental results, we characterize the nanoscale heated region from electromagnetic simulation of the tip‐substrate system. The heat generation rate within GaN substrate can be determined.^[^ ^39^ ^]^ The electric field distribution *E* of the tip‐substrate contact region is shown in Figure 2a. It is found that the enhanced electric field is confined to a small region with a radius of less than 10 nm underneath the nanotip. The intensity of this field can be significantly amplified by a factor of 79 due to the gold‐coated silicon nanotip. This enhanced electric field is further converted into nanoscale hotspots within the GaN substrate. For example, for the maximum laser power of 5.42 mW in experiments, the incident intensity (*I*_0_) is 1.9  ×  10^6^ W m^−2^ and the calculated distribution of heat generation rate *Q* in GaN is shown in Figure 2b. It illustrates that the hotspot size is also less than 10 nm, which is consistent with the hemi‐ellipsoidal distribution of the electric field. The lateral and vertical radii of the hotspot (i.e., the heat penetration) are *R_l_* = 7.2 nm and *R_v_* = 2.1 nm (the position at which the peak heat generation rate of the hotspot center drops to 1/*e*), respectively. It should be noted that determining the exact heat generation at such a nanoscale presents a significant challenge. The electromagnetic simulations represent a reasonable and effective method in the existing literature for calculating the spatial distribution of the excited nanoscale hotspots^[^ ^39^ ^]^ (see Supporting Information for details).

### 2.2 Theoretical Quantitative Predictions of Phonon Temperatures

To achieve a quantitative comparison, we further theoretically calculate phonon temperatures near the excited nanoscale hotspot under this tip‐enhanced Raman measurement setup using the phonon BTE combined with the first‐principles calculations. Although the multitemperature model has been widely employed to calculate phonon temperature nonequilibrium, it is based on diffusive transport theory that is not applicable for phonon transport near nanoscale hotspots.^[^ ^14^ ^]^ In contrast, the phonon BTE is the governing equation for phonon transport and scattering in solids at scales comparable to the phonon mean free path.^[^ ^42^, ^43^ ^]^ Mode‐level phonon temperatures can be determined by solving the phonon BTE. The steady‐state phonon BTE in the energy form is expressed as:^[^ ^44^ ^]^

(1) $$v_{\omega ,p}\cdot \nabla e_{\omega ,p,s}=\frac{e_{\omega ,p}^{0}-e_{\omega ,p,s}}{\tau_{\omega ,p}}+{\dot{q}}_{\omega ,p}.$$

*e_ω_*_,_ *_p_*_,_ **_s_** = *e*(**r**,**s**,*ω*,*p*) donates the volumetric energy density of phonons at position **r** in direction **s** with frequency *ω*, polarization *p*. $e_{\omega ,p}^{0}$ is the corresponding phonon equilibrium energy density. *τ_ω_*_,_ *_p_* and *v_ω_*_,_ *_p_* are the phonon relaxation time and group velocity, respectively. The heat generation term ${\dot{q}}_{\omega ,p}=\dot{q}\left(r,\omega ,p\right)$ is the energy transferred from electrons to the phonon mode (*ω*,*p*) in unit volume through electron‐phonon coupling. The summation of ${\dot{q}}_{\omega ,p}$ over phonon modes equals the total heat generation rate distribution *Q* of the excited hotspot as shown in Figure 2b. These mode‐level phonon properties needed for solving the phonon BTE can be accurately obtained from first‐principles calculations, relying solely on the input of the atomic structure^[45,^ ^46^ ^]^. Therefore, the first‐principles‐based phonon BTE does not necessitate any fitting parameters, and can accurately determine phonon temperature nonequilibrium at the nanoscale. However, performing first‐principles calculations and solving the high‐dimensional phonon BTE are challenging and entail considerable computational costs, which have significantly hindered quantitative investigations of nanoscale phonon transport in practical applications^[^ ^35^, ^47^ ^]^. Here, we have broken these limitations and developed an efficient solver for the complex first‐principles‐based phonon BTE to resolve phonon transport with spatial and momentum resolutions under nanoscale optical and electrical excitations in realistic 3D systems^[^ ^35^, ^36^ ^]^. Note that our calculations are set to be completely consistent with the scenario of heat conduction in the tip‐enhanced Raman measurement system to achieve a fair quantitative comparison, including the boundary conditions, the intensity, and the spatial distribution of the excited hotspot, etc. Further computational details are presented in the Experimental Section and Supporting Information.

The first‐principles‐based phonon BTE calculated results are shown in Figure 2c, which are found to agree quite well with the tip‐enhanced Raman thermal measurements across different laser powers. Note that the shown phonon temperatures in Figure 2c are Raman intensity weighted for the phonon temperature distribution near the nanoscale hotspot over the top surface of the GaN sample,^[^ ^27^ ^]^ which is consistent with the tip‐enhanced Raman measured temperatures. Significant differences are illustrated between the temperatures of different phonons, including the *A*_1_(LO) (lower triangle symbols) and the *E*_2_(TO) (upper triangle symbols) phonon modes, the average temperature of all the acoustic phonons (left triangle symbols), and also the lattice temperature (right triangle symbols), indicating a large phonon nonequilibrium near the nanoscale hotspot. In addition, the temperature rise of both *A*_1_(LO) and *E*_2_(TO) modes is significantly higher than the predictions from macroscale Fourier's law and the acoustic phonon modes. It should be noted that since no adjustable fitting parameters have been adopted in the first‐principles‐based phonon BTE calculations. The consistency between experimental measurements and theoretical calculations is a piece of strong evidence of phonon nonequilibrium induced in GaN at the nanoscale. To further show this phonon nonequilibrium, we present the calculated phonon temperature distribution near the excited nanoscale hotspot at the surface of the GaN sample in contact with the nanotip (i.e., the *z* = 0 plane) for incident laser power = 5.42 mW. As illustrated in Figure 2d, pronounced optical phonon temperature rises are excited, while the average acoustic phonon temperature rise is lower by nearly two orders of magnitude. Moreover, there is also a clear phonon temperature nonequilibrium between different optical phonons (e.g., *A*_1_(LO) and *E*_2_(TO) phonon modes, see Supporting Information for details).

### 2.3 Mechanisms of Nonequilibrium Phonon Transport

**Figure 3:**
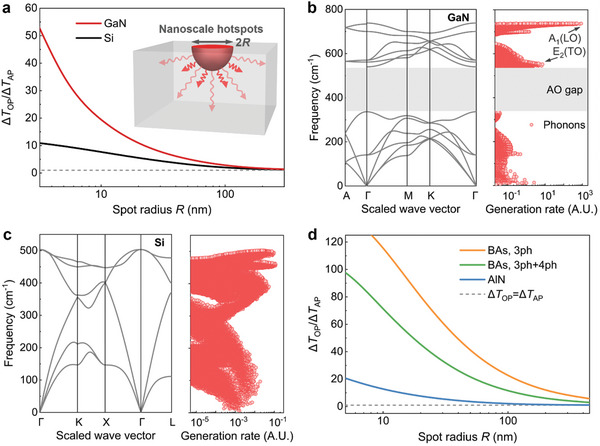
Mechanisms of nanoscale hotspot‐induced phonon nonequilibrium in GaN. a) The ratio of calculated temperature rise Δ*T*_OP_ to acoustic phonon temperature rise Δ*T*_AP_ within nanoscale hotspots in a bulk system shown in the inset for GaN and Si, which measures the magnitude of phonon nonequilibrium. The gray dashed line represents Δ*T*_OP_ = Δ*T*_AP_, i.e., no phonon nonequilibrium is introduced. The calculated phonon dispersion and phonon mode‐level heat generation rate for b) GaN and c) Si from first‐principles calculations are presented. Each red symbol corresponds to a certain phonon mode. The gray region shown in (**b**) represents the AO frequency gap in GaN, which is absent in Si. d) The ratio of Δ*T*_OP_ to Δ*T*_AP_ within the hotspot shown in a) for AlN and BAs. Both three‐phonon and four‐phonon processes are considered for BAs.

We further explore the underlying physical mechanisms of such substantial phonon nonequilibrium induced in GaN by analyzing a simple nanoscale hotspot as shown in **Figure** **3**a. A 3D bulk system is considered, in which a hemispherical nanoscale hotspot with a radius of *R* and uniform heat generation within it is located on the top surface. The top surface is considered adiabatic and the generated heat in the hotspot is transferred to the heat sink in other directions. Note that this hotspot is different from that in the tip‐enhanced Raman measurements, which can provide a clearer illustration of the underlying physical mechanisms of the induced phonon nonequilibrium. We aim to calculate the phonon temperature nonequilibrium in GaN by comparing that in Si near the same nanoscale hotspots using first‐principles‐based phonon BTE calculations (see the Experimental Section for details). The ratio of the calculated average optical phonon temperature rise ∆*T*_OP_ to the average acoustic phonon temperature rise ∆*T*_AP_ within the hotspot as a function of the hotspot radius is shown in Figure 3a, which represents the magnitude of induced phonon nonequilibrium.^[^ ^28^ ^]^ The results reveal that clear phonon nonequilibrium is introduced in both GaN and Si as the hotspot scales down to several hundred nanometers, and increases rapidly with decreasing hotspot radius. Again, an unusually large phonon nonequilibrium is observed in GaN, distinguishing it from Si, wherein the optical phonon temperature rise is tens of times higher than the acoustic phonon temperature rise as the hotspot shrinks to the sub‐10 nm regime.

To illustrate this distinction in phonon nonequilibrium between GaN and Si, we further examine the detailed phonon transport properties. As shown in Figure 3b,c, the phonon dispersion and the phonon mode‐level heat generation rate (i.e., the energy transfer rate from electrons to phonons) from first‐principles calculations are illustrated. The results demonstrate two principal differences between GaN and Si. First, GaN shows an evident peak of heat generation rate concentrated at the maximum phonon frequency which corresponds to LO phonon modes near the Brillion zone center. However, for Si, although the optical phonons still obtain more energy from electrons, the heat generation rate is relatively uniformly distributed across different phonon modes. Our calculations show that ≈94% of the total energy transferred from electrons to phonons in GaN is received by phonon modes within the top LO branch shown in Figure 3b. It indicates a strong coupling between electrons and high‐frequency LO phonons, while the coupling with other phonons is significantly weaker. This phenomenon of strong and selective electron‐phonon coupling results from the fact that the fluctuations of the ionic positions (LO phonons) in a polar material generate a macroscopic electric field that can intensely interact with electrons and holes, known as the Fröhlich electron‐phonon coupling^[^ ^48^ ^]^ (see Supporting Information). This highly selective electron‐phonon coupling leads to highly selective phonon excitation in GaN shown in Figure 3b, which can directly cause large temperature differences among different phonons.^[^ ^18^ ^]^ As marked in Figure 3b, the *A*_1_(LO) phonon mode obtains more energy from electrons than both the *E*_2_(TO) phonon mode and acoustic phonons, which explains its higher temperature rise as presented in Figure 2c. Second, there is a large AO frequency gap in the phonon dispersion of GaN as shown by the gray region in Figure 3b. It arises from the large mass difference between constituent atoms (i.e., the gallium atom and the nitride atom) and is absent in Si. This large AO frequency gap weakens the interaction between optical and acoustic phonons due to the difficulty in satisfying momentum and energy conservation simultaneously.^[^ ^49^ ^]^ As a result, acoustic phonons tend to transport ballistically near the hotspot with rare phonon‐phonon scattering. Therefore, hot optical phonons cannot efficiently transfer the large amounts of energy obtained from electrons to acoustic phonons, causing even more significant temperature differences between optical and acoustic phonons. Consequently, the negligible energy obtained from electrons for acoustic phonons and their weak coupling with hot optical phonons lead to elevated optical phonon temperatures and orders of magnitude lower acoustic phonon temperatures as presented in Figure 2c and Figure 3a. We emphasize that it is the combination of these two mechanisms that leads to the substantial phonon temperature nonequilibrium in GaN.

## 3 Discussion

The two mechanisms for the substantial phonon nonequilibrium in GaN elucidated above, i.e., the strong Fröhlich electron‐phonon coupling and large AO frequency gap, originate from the large electronegativity difference and large mass difference between constituent atoms. It is worth noting that these two origins are common features in some III‐V polar semiconductors. For example, the AlN also has a significant electronegativity difference and the BAs has an even larger mass difference between constituent atoms than that of GaN, which are also promising semiconductor materials in nanodevices.^[^ ^50^, ^51^ ^]^ To further illustrate the impact of these two mechanisms on phonon nonequilibrium in 3D semiconductors, we implement the first‐principles‐based phonon BTE calculations for AlN and BAs under the case shown in the inset of Figure 3a. Previous investigations have demonstrated the significant role of four‐phonon processes in some III‐V semiconductors, especially for BAs.^[^ ^52^ ^]^ Therefore, the four‐phonon scattering rate has been considered in our first‐principles calculations for BAs but is ignored for GaN and AlN because of the negligible contributions as demonstrated in previous literature.^[^ ^53^ ^]^ The results for the ratio of average optical to acoustic phonon temperature rises within nanoscale hotspots are presented in Figure 3d. As expected, large phonon nonequilibrium is also induced in AlN and BAs due to the concentrated heat generation rate for optical phonons and weak coupling between optical and acoustic phonons (see Supporting Information for details). Moreover, it indicates that four‐phonon scattering in BAs weakens the magnitude of nonequilibrium between optical and acoustic phonons. This is because the significant four‐phonon processes facilitate additional phonon‐phonon scattering events and frequent energy exchange between hot optical phonons and acoustic phonons, consequently diminishing the temperature difference between them.

It is noteworthy that this study presents a direct evidence of significant phonon nonequilibrium at the sub‐10 nm regime in realistic 3D device semiconductors. For modern Si‐based nanodevices, such as fin field‐effect transistors, the device size has been scaled down to 10 nm,^[^ ^9^ ^]^ with even smaller nanoscale hotspots generated in the near‐junction region of the device. These nanoscale hotspots can lead to large phonon nonequilibrium and thus dramatically elevated temperature rises,^[^ ^18^ ^]^ posing significant challenges on the electrical performance and reliability of nanodevices. Although devices based on III‐V semiconductors are typically power electronics (such as GaN high electron mobility transistors) with relatively large device size, the generated hotspots can shrink to the sub‐microscale.^[^ ^24^ ^]^ Considering that III‐V semiconductors induce more pronounced phonon nonequilibrium than Si as discussed in Figure 3, the deterioration in thermal transport within devices based on III‐V semiconductors can be even more severe. Therefore, this study reveals a viable way to enhance the thermal transport in nanoscale devices, i.e., reducing the magnitude of phonon nonequilibrium. A recent work published during the peer review process of this study has demonstrated the effectiveness of reducing temperature rise through minimizing the directional phonon nonequilibrium by adding defects in nanoscale hotspots.^[^ ^54^ ^]^

## 4 Conclusion

In summary, we present experimentally and theoretically unified evidence of phonon nonequilibrium near nanoscale hotspots in realistic 3D device semiconductors. A tip‐enhanced Raman thermal measurement is designed and directly reveals substantial phonon temperature nonequilibrium near sub‐10 nm laser spots in GaN, which is further demonstrated by quantitative agreements between measurements and the first‐principles‐based phonon BTE calculations. We illustrate that this unusually pronounced phonon nonequilibrium originated from the highly selective phonon excitation due to strong Fröhlich electron‐phonon coupling, and the weak optical‐acoustic phonon coupling caused by the large AO frequency gap in GaN. This large phonon nonequilibrium is also demonstrated to be a universal phenomenon in 3D materials at the nanoscale, especially for III‐V polar semiconductors. Our work establishes a viable approach for investigating phonon nonequilibrium at the nanoscale, which can provide deep insights into the intricate nonequilibrium dynamics of energy carriers in nanoelectronics.

## 5 Experimental Section

### Tip‐Enhanced Raman Thermal Experiments

In the tip‐enhanced Raman thermal experiments, a polarized 532 nm laser, inclined at an incident angle of less than 15° relative to the GaN surface, was applied to focus on and heat the silicon nanotip coated with gold of an AFM instrument. Positioned beneath and in contact with this tip was a bulk GaN sample, giving rise to the creation of a highly localized heated region underneath the tip, as shown in Figure 1a. The selection of an ≈15° incident angle for the laser beam to the substrate ensures significant electric field enhancement,^[^ ^55^ ^]^ generates a relatively strong Raman signal, and remains practical for implementation in the experimental setup. The AFM tip (ScanSens, CSG01 series model) shows a half angle of θ = 10° and apex radius of *r*_1_ = 30 nm. It was coated with a 20 nm‐thick layer of gold. The dimensions of the GaN sample were measured at 4.23  ×  2.19  ×  0.64 cm^3^. Raman peak shifts of the *E*_2_(TO) and *A*_1_(LO) phonon modes were used to quantify their temperature rises. The detailed setup of the optical alignment for thermal sensing using tip‐enhanced Raman is presented in Figure 1a. The Raman probing lens was fixed on a three‐axis translation stage, enabling precise movement of the laser beam within the confined target area. The focused laser spot through the objective was ≈60 µm in diameter. The position of the Raman probing lens was adjusted to focus the laser on the tip apex and heat the GaN substrate. Both the spectrometer and the laser generator were interfaced with a computer and controlled through a pre‐installed software. Feedback signals were received and processed through a program integrated into the support software. To characterize the phonon temperature rises in GaN, calibration experiments were carried out to analyze the linear relationship between temperature and Raman peak frequency (see Supporting Information for details). Notably, the quantified phonon temperature rises from Raman peak shifts were within the temperature range of calibration experiments.

### First‐Principles‐Based Phonon BTE Calculations

In the phonon BTE given by Equation (1), *e_ω_*_,_ *_p_*_,_ **_s_** = *e*(**r**,**s**,*ω*,*p*) = *ћωD_p_* (*ω*)*n*(**r**,**s**,*ω*,*p*) donates the volumetric energy density of phonons at position **r** in direction **s** with frequency *ω*, polarization *p*, and wave vector **q**, where *n = n*(**r**,**s**,*ω*,*p*) is the phonon distribution function. The equivalent phonon temperature *T_ω_*_,_ *_p_* could be obtained from the summation of energy density over phonons with frequency *ω* and polarization *p* according to $T_{\omega ,p}=\int_{\Omega^{\prime }}\frac{e_{\omega ,p,s}}{C_{\omega ,p}}d\Omega^{\prime }$,^[^ ^56^, ^57^ ^]^ where *C_ω_*_,_ *_p_* is the phonon heat capacity. The left‐hand side of Equation (1) is the phonon transport term, while the right‐hand side is the scattering term that is dominated by phonon‐phonon scattering, phonon‐impurity scattering, and electron‐phonon scattering in the bulk system under optical/electric excitations.^[^ ^46^ ^]^ The phonon‐phonon scattering and phonon‐impurity scattering (only considering isotope scattering^[^ ^58^ ^]^) were usually described by the relaxation time approximation,^[^ ^59^ ^]^ and the electron‐phonon scattering could be reduced to a mode‐level energy transfer rate term ${\dot{s}}_{\omega ,p}$.^[^ ^60^ ^]^ This ${\dot{s}}_{\omega ,p}$ is the energy transferred from electrons to the phonon mode in unit volume and unit time at a certain equivalent electron temperature *T*_e_, as determined by the Fermi golden rule:^[^ ^10^ ^]^

(2) $$\begin{array}{ccc} {\dot{s}}_{\omega ,p} & = & \frac{4\pi }{\hbar V}\sum_{k}{\hbar \omega \left|M^{nmp}\left(k,q\right)\right|}^{2}\left\{n_{qp}\left(f_{mk+q}\left(T_{e}\right)-f_{nk}\left(T_{e}\right)\right)\right.\\ & & \left.+\;f_{mk+q}\left(T_{e}\right)\left(1-f_{nk}\left(T_{e}\right)\right)\right\}\delta \left(E_{nk}-E_{mk+q}+\hbar \omega \right). \end{array}$$

Here, (*n*, *m*) are the band indices; **k** is the wave vector of electrons with the distribution function *f*; *M* is the electron‐phonon coupling matrix. The local electrons were assumed in the equilibrium Fermi‐Dirac distribution with an equivalent temperature *T*_e_ in the first‐principles calculations, which could be determined from the total energy power density applied by the incident Raman laser in experiments.^[^ ^10^ ^]^ The Fröhlich coupling of electrons with LO phonons was rigorously addressed within the framework of our first‐principles calculations. Note that it was the proportion of energy obtained by different phonon modes that matters, i.e., $\frac{{\dot{s}}_{\omega ,p}}{\sum_{\omega ,p}{\dot{s}}_{\omega ,p}}$. The actual phonon mode‐level heat generation rate was then determined by ${\dot{q}}_{\omega ,p}=\frac{{\dot{s}}_{\omega ,p}}{\sum_{\omega ,p}{\dot{s}}_{\omega ,p}}Q$, where *Q* is the total heat generation rate distribution from electromagnetic simulations (see Supporting Information for details). This study had also found that the electron equivalent temperature/carrier concentration had a negligible effect on the proportion of energy obtained by different phonon modes (Figure S3, Supporting Information). All these mode‐level phonon properties were accurately resolved using first‐principles calculations based on QUANTUM‐ESPRESSO,^[^ ^61^, ^62^ ^]^ ShengBTE,^[^ ^63^ ^]^ and FourPhonon^[^ ^64^ ^]^ packages. Taking these phonon properties as input, the phonon BTE (Equation (1)) could be numerically solved to calculate phonon temperatures using a recently developed efficient solver by our group.^[^ ^36^ ^]^ Details of the first‐principles‐based phonon BTE calculations are provided in Supporting Information.

## Conflict of Interest

The authors declare no conflict of interest.

## Author Contributions

H.B. and Y.Y. conceived the research. J.X. and Y.S. designed and carried out the first‐principle‐based phonon BTE calculations. X.H., Q.S., and H.Z. customized and performed the tip‐enhanced Raman thermal measurements and electromagnetic simulations. J.X. and X.H. analyzed the data, interpreted the results and prepared the figures and the manuscript. H.B. and Y.Y. supervised the research.

## Supporting information



Supporting Information

## Data Availability

The data that support the findings of this study are available from the corresponding author upon reasonable request.

## References

[1] R. Chau , B. Doyle , S. Datta , J. Kavalieros , K. Zhang , Nat. Mater. 2007, 6, 810. 17972935 10.1038/nmat2014

[2] J. A. del Alamo , Nature 2011, 479, 317. 22094691 10.1038/nature10677

[3] Y. D. Kim , H. Kim , Y. Cho , J. H. Ryoo , C.‐H. Park , P. Kim , Y. S. Kim , S. Lee , Y. Li , S.‐N. Park , Y. Shim Yoo , D. Yoon , V. E. Dorgan , E. Pop , T. F. Heinz , J. Hone , S.‐H. Chun , H. Cheong , S. W. Lee , M.‐H. Bae , Y. D. Park , Nat. Nanotechnol. 2015, 10, 676. 26076467 10.1038/nnano.2015.118

[4] S. Volz , J. Ordonez‐Miranda , A. Shchepetov , M. Prunnila , J. Ahopelto , T. Pezeril , G. Vaudel , V. Gusev , P. Ruello , E. M. Weig , M. Schubert , M. Hettich , M. Grossman , T. Dekorsy , F. Alzina , B. Graczykowski , E. Chavez‐Angel , J. Sebastian Reparaz , M. R. Wagner , C. M. Sotomayor‐Torres , S. Xiong , S. Neogi , D. Donadio , Eur. Phys. J. 2016, 89, 15.

[5] Q. Weng , S. Komiyama , L. Yang , Z. An , P. Chen , S.‐A. Biehs , Y. Kajihara , W. Lu , Science 2018, 360, 775. 29599192 10.1126/science.aam9991

[6] V. A. Jhalani , J.‐J. Zhou , M. Bernardi , Nano Lett. 2017, 17, 5012. 28737402 10.1021/acs.nanolett.7b02212

[7] H. Xue , R. Qian , W. Lu , X. Gong , L. Qin , Z. Zhong , Z. An , L. Chen , W. Lu , Nat. Commun. 2023, 14, 3731. 37349328 10.1038/s41467-023-39489-z PMC10287675

[8] J. Zhou , H. D. Shin , K. Chen , B. Song , R. A. Duncan , Q. Xu , A. A. Maznev , K. A. Nelson , G. Chen , Nat. Commun. 2020, 11, 6040. 33247148 10.1038/s41467-020-19938-9 PMC7695728

[9] E. Pop , Nano Res. 2010, 3, 147.

[10] E. Minamitani , Phy. Rev. B 2021, 104, 085202.

[11] S. Sadasivam , M. K. Y. Chan , P. Darancet , Phys. Rev. Lett. 2017, 119, 136602. 29341683 10.1103/PhysRevLett.119.136602

[12] G. Chen , J. Heat Mass Transf. Res. 1996, 118, 539.

[13] X. Tong , M. Bernardi , Phys. Rev. Res. 2021, 3, 023072.

[14] A. K. Vallabhaneni , D. Singh , H. Bao , J. Murthy , X. Ruan , Phys. Rev. B 2016, 93, 125432.

[15] M. An , Q. Song , X. Yu , H. Meng , D. Ma , R. Li , Z. Jin , B. Huang , N. Yang , Nano Lett. 2017, 17, 5805. 28777582 10.1021/acs.nanolett.7b02926

[16] V. Chiloyan , S. Huberman , A. A. Maznev , K. A. Nelson , G. Chen , Appl. Phys. Lett. 2020, 116, 163102.

[17] T. Feng , W. Yao , Z. Wang , J. Shi , C. Li , B. Cao , X. Ruan , Phys. Rev. B 2017, 95, 195202.

[18] J. Xu , Y. Hu , H. Bao , Phys. Rev. Appl. 2023, 19, 014007.

[19] Q. Weng , L. Yang , Z. An , P. Chen , A. Tzalenchuk , W. Lu , S. Komiyama , Nat. Commun. 2021, 12, 4752. 34362908 10.1038/s41467-021-25094-5 PMC8346506

[20] F. Sekiguchi , H. Hirori , G. Yumoto , A. Shimazaki , T. Nakamura , A. Wakamiya , Y. Kanemitsu , Phys. Rev. Lett. 2021, 126, 077401. 33666485 10.1103/PhysRevLett.126.077401

[21] J. Yang , X. Wen , H. Xia , R. Sheng , Q. Ma , J. Kim , P. Tapping , T. Harada , T. W. Kee , F. Huang , Y.‐B. Cheng , M. Green , A. Ho‐Baillie , S. Huang , S. Shrestha , R. Patterson , G. Conibeer , Nat. Commun. 2017, 8, 14120. 28106061 10.1038/ncomms14120 PMC5263885

[22] Z. Lu , A. Vallabhaneni , B. Cao , X. Ruan , Phys. Rev. B 2018, 98, 134309.

[23] Y. Hu , L. Zeng , A. J. Minnich , M. S. Dresselhaus , G. Chen , Nat. Nanotechnol. 2015, 10, 701. 26030656 10.1038/nnano.2015.109

[24] G. Chen , B. Hu , Z. Wang , D. Tang , Int. J. Therm. Sci. 2023, 194, 108592.

[25] R. Li , E. Lee , T. Luo , Mater. Today Phys. 2021, 19, 100429.

[26] Y. Shen , X. S. Chen , Y. C. Hua , H. L. Li , L. Wei , B. Y. Cao , IEEE Trans. Electron Devices 2023, 70, 409.

[27] W. Cai , A. L. Moore , Y. Zhu , X. Li , S. Chen , L. Shi , R. S. Ruoff , Nano Lett. 2010, 10, 1645. 20405895 10.1021/nl9041966

[28] R. Wang , H. Zobeiri , Y. Xie , X. Wang , X. Zhang , Y. Yue , Adv. Sci. 2020, 7, 2000097. 10.1002/advs.202000097 PMC7341092 32670758

[29] S. Sullivan , A. Vallabhaneni , I. Kholmanov , X. Ruan , J. Murthy , L. Shi , Nano Lett. 2017, 17, 2049. 28218545 10.1021/acs.nanolett.7b00110

[30] H. Zobeiri , N. Hunter , R. Wang , T. Wang , X. Wang , Adv. Sci. 2021, 8, 2004712. 10.1002/advs.202004712 PMC8224447 34194932

[31] N. Hunter , N. Azam , H. Zobeiri , N. Van Velson , M. Mahjouri‐Samani , X. Wang , Adv. Mater. Interfaces 2022, 9, 2102059.

[32] L. Lindsay , D. A. Broido , N. Mingo , Phys. Rev. B 2010, 82, 115427.

[33] Q. Guo , D. Li , Q. Hua , K. Ji , W. Sun , W. Hu , Z. L. Wang , Nano Lett. 2021, 21, 4062. 33885320 10.1021/acs.nanolett.1c00999

[34] M. Parker , Nat. Electron. 2021, 4, 858.

[35] Y. Hu , Y. Shen , H. Bao , Fundamental Research 2024, 4, 907. 10.1016/j.fmre.2022.06.007 PMC11330117 39156572

[36] Y. Hu , R. Jia , J. Xu , Y. Sheng , M. Wen , J. Lin , Y. Shen , H. Bao , J. Phys.: Condens. Matter 2024, 36, 025901. 10.1088/1361-648X/acfdea 37757854

[37] Z. Zheng , L. Zhang , W. Song , S. Feng , H. Xu , J. Sun , S. Yang , T. Chen , J. Wei , K. J. Chen , Nat. Electron. 2021, 4, 595.

[38] D.‐S. Tang , B.‐Y. Cao , Int. J. Heat Mass Transfer 2023, 200, 123497.

[39] D. Huang , Q. Sun , Z. Liu , S. Xu , R. Yang , Y. Yue , Adv. Sci. 2023, 10, 2204777. 10.1002/advs.202204777 PMC9839872 36394164

[40] Y. Yue , X. Chen , X. Wang , ACS Nano 2011, 5, 4466. 21557563 10.1021/nn2011442

[41] V. Y. Davydov , Y. E. Kitaev , I. N. Goncharuk , A. N. Smirnov , J. Graul , O. Semchinova , D. Uffmann , M. B. Smirnov , A. P. Mirgorodsky , R. A. Evarestov , Phys. Rev. B 1998, 58, 12899.

[42] J. M. Ziman , Electrons and Phonons: the Theory of Transport Phenomena in Solids, Oxford University Press, Oxford, UK 2001.

[43] M. Kaviany , Heat Transfer Physics, Cambridge University Press, Cambridge, UK 2014.

[44] J. M. Loy , J. Y. Murthy , D. Singh , J. Heat Transfer 2012, 135, 011008.

[45] D. A. Broido , M. Malorny , G. Birner , N. Mingo , D. A. Stewart , Appl. Phys. Lett. 2007, 91, 231922.

[46] H. Bao , J. Chen , X. Gu , B. Cao , ES Energy Environ. 2018, 1, 16.

[47] S. A. Ali , G. Kollu , S. Mazumder , P. Sadayappan , A. Mittal , Int. J. Therm. Sci. 2014, 86, 341.

[48] C. Verdi , F. Giustino , Phys. Rev. Lett. 2015, 115, 176401. 26551127 10.1103/PhysRevLett.115.176401

[49] N. K. Ravichandran , D. Broido , Phys. Rev. X 2020, 10, 021063.

[50] G. Greco , P. Fiorenza , F. Iucolano , A. Severino , F. Giannazzo , F. Roccaforte , ACS Appl. Mater. Interfaces 2017, 9, 35383. 28920438 10.1021/acsami.7b08935

[51] J. S. Kang , M. Li , H. Wu , H. Nguyen , Y. Hu , Science 2018, 361, 575. 29976798 10.1126/science.aat5522

[52] T. Feng , L. Lindsay , X. Ruan , Phys. Rev. B 2017, 96, 161201.

[53] X. Yang , T. Feng , J. Li , X. Ruan , Phys. Rev. B 2019, 100, 245203.

[54] Y. Hu , J. Xu , X. Ruan , H. Bao , Nat. Commun. 2024, 15, 3304. 38632242 10.1038/s41467-024-47716-4 PMC11024214

[55] Y. Chen , Y. Xu , D. Xie , J. Jiang , Y. Yue , Appl. Therm. Eng. 2019, 148, 129.

[56] Y. Hu , T. Feng , X. Gu , Z. Fan , X. Wang , M. Lundstrom , S. S. Shrestha , H. Bao , Phys. Rev. B 2020, 101, 155308.

[57] J. Xu , Y. Hu , X. Ruan , X. Wang , T. Feng , H. Bao , Phys. Rev. B 2021, 104, 104310.

[58] M. Berglund , M. E. Wieser , Pure Appl. Chem. 2011, 83, 397.

[59] A. Majumdar , J. Heat Transfer. 1993, 115, 7.

[60] E. Pop , S. Sinha , K. E. Goodson , Proc. IEEE. 2006, 94, 1587.

[61] P. Giannozzi , S. Baroni , N. Bonini , M. Calandra , R. Car , C. Cavazzoni , D. Ceresoli , G. L. Chiarotti , M. Cococcioni , I. Dabo , A. Dal Corso , S. de Gironcoli , S. Fabris , G. Fratesi , R. Gebauer , U. Gerstmann , C. Gougoussis , A. Kokalj , M. Lazzeri , L. Martin‐Samos , N. Marzari , F. Mauri , R. Mazzarello , S. Paolini , A. Pasquarello , L. Paulatto , C. Sbraccia , S. Scandolo , G. Sclauzero , A. P. Seitsonen , et al., J. Phys.: Condens. Matter 2009, 21, 395502. 21832390 10.1088/0953-8984/21/39/395502

[62] S. Poncé , E. R. Margine , C. Verdi , F. Giustino , Comput. Phys. Commun. 2016, 209, 116.

[63] W. Li , J. Carrete , N. A. Katcho , N. Mingo , Comput. Phys. Commun. 2014, 185, 1747.

[64] Z. Han , X. Yang , W. Li , T. Feng , X. Ruan , Comput. Phys. Commun. 2022, 270, 108179.

