# Supplementary material for: Direct Observation of Substantial Phonon Nonequilibrium Near Nanoscale Hotspots in Gallium Nitride
Source: Adv Sci (Weinh). 2025 Jan 24;12(11):2411040. doi: 10.1002/advs.202411040 (PMC11924035; doi:10.1002/advs.202411040)
Supplement: Supplementary file 1 — Supporting Information [file ADVS-12-2411040-s001.docx]

Supporting Information for

**Direct observation of substantial phonon nonequilibrium near nanoscale hotspots in gallium nitride**

Jiaxuan Xu^1,2^†, Xiaona Huang^1^†, Yufei Sheng^2^, Qiangsheng Sun^1^, Hongkai Zhang^1^, Hua Bao^3^*, Yanan Yue^1,4^*

*^1^School of Power and Mechanical Engineering, Wuhan University, Wuhan, Hubei 430072, People’s Republic of China.*

*^2^University of Michigan-Shanghai Jiao Tong University Joint Institute, Shanghai Jiao Tong University, Shanghai 200240, People’s Republic of China.*

*^3^Global Institute of Future Technology, Shanghai Jiao Tong University, Shanghai 200240, People’s Republic of China.*

*^4^Department of Mechanical and Manufacturing Engineering, Miami University, Oxford, Ohio 45056, United States*

*Corresponding authors. E-mails: hua.bao@sjtu.edu.cn; yyue@whu.edu.cn.

†These authors contributed equally to this work.

**Section S1. Details of tip-enhanced Raman thermal experiments**

**Section S2. Raman calibration experiments**

**Section S3. Electromagnetic simulation and thermal analysis of the tip-substrate system**

**Section S4. Details of first-principles calculations and phonon BTE calculations**

**Section S5. Phonon temperature distributions**

**Section S6. Phonon dispersion and mode-level heat generation rate for AlN and BAs**

# S1. Details of tip-enhanced Raman thermal experiments

Fig. S1 shows the setup of the tip-enhanced Raman thermal experiments. A polarized 532 nm laser, inclined at an incident angle of around 15° relative to the GaN surface, is applied to focus on and heat the silicon nanotip coated with gold of an AFM instrument. Positioned beneath and in contact with this tip is a bulk GaN sample, giving rise to the creation of a highly localized heated region underneath the tip, as shown in Fig. 1a in the main text. The AFM tip (ScanSens, CSG01 series model) shows a half angle of $\theta$ = 10° and apex radius of *r*_1_ = 30 nm. It is coated with a 20 nm-thick layer of gold. The nanotip apex configuration used in our experiments is a composite structure, where the tip’s sharp end is tangential to a hemisphere, as shown in Fig. S2. The dimensions of the GaN sample are measured at 4.23 × 2.19 × 0.64 cm^3^. The detailed setup of the optical alignment for thermal sensing using tip-enhanced Raman is presented in Fig. 1a in the main text. The Raman probing lens (50×) is ﬁxed on a three-axis translation stage, enabling precise movement of the laser beam within the confined target area. The focused laser spot through the objective is ~60 μm in diameter. The position of the Raman probing lens is adjusted to focus the laser on the tip apex and heat the GaN substrate. Specifically, the probing lens is first moved horizontally along the direction of the cantilever. Once the strongest Rayleigh scattering intensity is observed, the laser is confirmed to be focused at the tip center. Subsequently, the lens is adjusted vertically to the spot where the Raman signal is just detected. Both the spectrometer and the laser generator are interfaced with a computer and controlled through a pre-installed software. Feedback signals are received and processed through a program integrated into the support software. Raman peak shifts of the *E*_2_(TO) and *A*_1_(LO) phonon modes are used to quantify their temperature rises. The gratings of 1800 grooves/mm with a spectral resolution of 0.44 cm^-1^ is utilized. The Raman shift of each peak is obtained by fitting the peak using the Lorentz function. Specifically, the Raman shift of the *E*_2_(TO) peak is determined by two-peak fitting using the Lorentz function. Three spectra are collected at each measurement and averaged to reduce measurement uncertainty.

To characterize the phonon temperature rises in GaN, calibration experiments are carried out to analyze the linear relationship between temperature and Raman shift (see Sec. S2). The measured Raman spectrum of the GaN from tip-enhanced experiments is shown in Fig. S3. A previous study showed that *E*_2_(TO) and *A*_1_(LO) phonons are allowed in the backscattering geometry from the standard *c*-plane, while E1 mode is observed only in a crossed polarization geometry [*1, 2*]. In our experiments, an intermediate propagation direction (around 15° relative to the GaN surface) was used, allowing for the simultaneous observation of both *E*_1_(TO) and *E*_2_(TO) phonons. The Raman intensity peaks of *E*_2_(TO) and *A*_1_(LO) phonon modes under the laser power from 2.61 to 5.42 mW are used to characterized the average phonon temperature rise in the heated region as shown in Fig. S4. It is noteworthy that the *A*_1_(LO) mode exhibits significantly weaker intensity compared to the *E*_2_(TO) mode. Consequently, the *A*_1_(LO) mode requires significantly long integration time to improve the signal-to-noise ratio and obtain reliable Raman signals. Additional experiments are conducted to acquire the *A*_1_(LO) mode signals at the lowest and highest power points using a long accumulation time. As shown in Fig. 2c in the main text, the maximum error in the temperature rise is 2.1 K in the *E*_2_(TO) mode and 7.3 K in the *A*_1_(LO) mode, primarily due to the relatively weak intensity leading to large uncertainty in the *A*_1_(LO) mode.

Furthermore, the small incident angle of the laser (around 15º) relative to the substrate surface, combined with the low absorptivity of GaN (532 nm in laser wavelength), renders the direct laser heating of the substrate negligible. To verify this, we also conduct an experiment on the GaN substrate without the nanotip. The Raman shifts of the phonon modes under various laser powers are shown in Fig. S5. The observed temperature rises were less than 1 K, significantly lower than the temperature characterized in the presence of the nanotip.


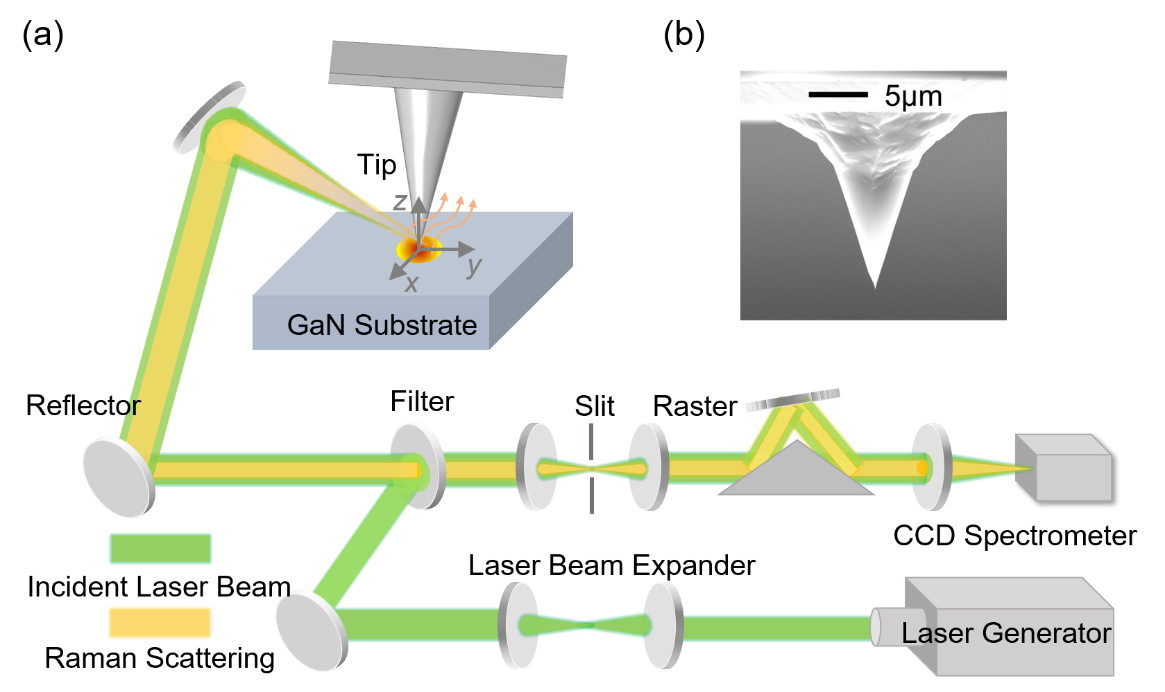


# Fig. S1

(a) Schematic diagram of optical alignment for tip-enhancement Raman measurements. (b) The SEM image of the gold coated silicon nanotip with a half-cone angle of 20°.


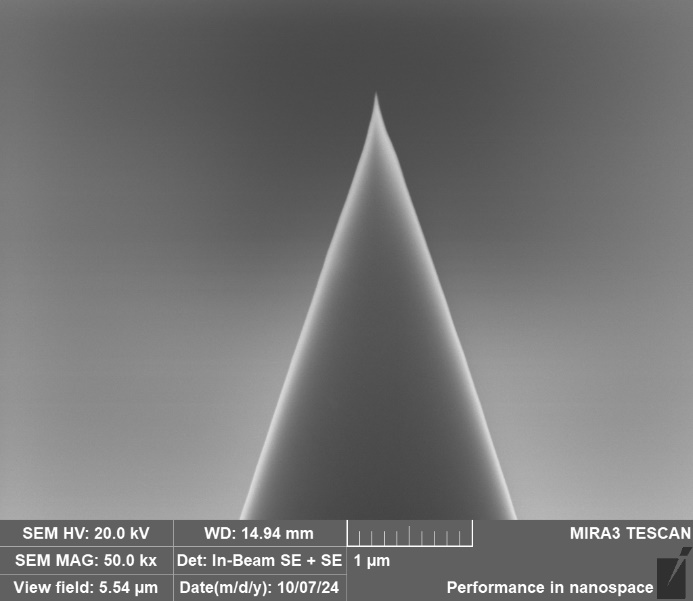


**Fig. S2.**

An SEM image of the tip apex.





# Fig. S3

The measured Raman spectrum of the GaN from tip-enhanced Raman experiments.





**Fig. S4**

Raman shifts of the *E*_2_(TO) and *A*_1_(LO) phonon modes in GaN under different incident laser powers measured with the laser beam focused on the nanotip apex.





**Fig. S5**

Raman shifts of the *E*_2_(TO) and *A*_1_(LO) phonon modes in GaN under different incident laser powers measured during direct laser heating without the nanotip.

# S2. Raman calibration experiments

The temperature rise in GaN is determined based on the linear relationship between temperature and Raman peak frequency. Thus, calibration experiments are carried out to analyze the linear relationship. The sample is placed on a heating stage to heat it at diﬀerent temperatures and the corresponding Raman spectra are recorded. As illustrated in Fig. S6, the Raman shift corresponding to *E*_2_(TO) and *A*_1_(LO) modes in GaN shows a well-defined linear association with temperature. This relation can be expressed as *ω*=*C_s_*(*T_ω_* - *T*_0_) + *ω*_0_, where *ω* represents the measured Raman shift, *T_ω_* denotes the sample temperature, *C_s_* is a calibration constant to be determined, and *ω*_0_ is the Raman shift at room temperature. The temperature coeﬃcients of *E*_2_(TO) and *A*_1_(LO) modes during its calibration are fitted as −0.014 cm^−1^ K^−1^ and −0.034 cm^−1^ K^−1^ respectively, which are in good agreement with previously reported data [*3, 4*]. Notably, the quantified phonon temperature rises from Raman peak shifts are within the temperature range of calibration experiments.


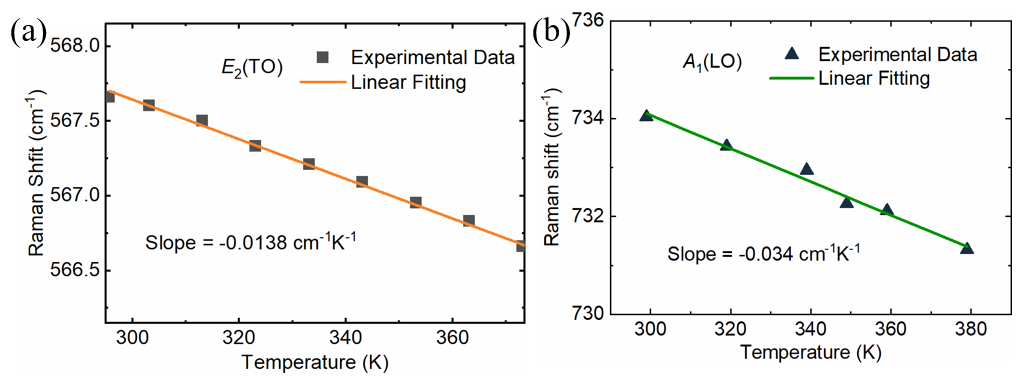


# Fig. S6

The linear relationship between Raman peak positions (**a**) *E*_2_(TO) and (**b**) *A*_1_(LO) phonon modes with temperature in GaN substrate.

# S3. Electromagnetic simulation and thermal analysis of the tip-substrate system

The electric field distribution of the tip-substrate system is simulated and the heat generation rate of the hotspot in GaN is analyzed. The gold-coated silicon nanotip and GaN substrate system are irradiated by a polarized 532 nm laser beam, inclined at an incident angle of 20° relative to the GaN surface. The silicon tip is coated with a 20 nm-thick layer of gold, and beneath it lays a 100 nm-thick GaN substrate. Consistent with our experiments, the coated tip features a half taper angle of 𝜃 = 10°, an apex radius of *r*_1_ = 30 nm, *r*_2_ = 50 nm, and a length of *L* = 300 nm. A rectangular computational domain of 600 × 600 × 800 nm^3^ is considered, containing the nanotip, substrate, and vacuum region around them. The electric field distribution within this domain is calculated by solving Maxwell’s equations using the finite difference time domain (FDTD) method. The perfectly matched layer (PML) absorbing boundary condition is applied, and the incident electric vector *E*_0_ is set to 1 V/m. The functions for gold and GaN are $\epsilon$_Au_ = −4.2854 + 2.3292i and $\epsilon$_GaN_ = 5.8254 – 0.4070i at 532 nm [*5*], respectively. Fig. 2a in the main text displays the cross-section view of the electric field distribution in the tip-substrate system. A strongly enhanced electric field is observed near the tip-substrate contact area, whereas other areas exhibit comparatively lower electric field enhancements. This significant field enhancement likely emanates from the effective exciting of the tip, where free electrons are confined at the tip apex, and surface plasmon resonance is induced by the gold coating.

The converted thermal energy of the hotspot in GaN is further calculated based on the above electric field distribution and Poynting’s theorem. The localized heat generation rate for the medium absorbing passed light can be calculated as *q*_loc_ = 0.5𝜖_0_𝜔Im[𝜖(𝜔)]*E*_loc_^2^, where 𝜖_0_, 𝜔, and *E*_loc_ are the vacuum permittivity, the incident wave angular frequency, and the normalized local electric ﬁeld, respectively [*6*]. Im[𝜖(𝜔)] is the imaginary part of the dielectric function of GaN at the incident wave frequency. Taking the maximum laser power of 5.42 mW as an example, the incident intensity *I*_0_ = 1.9 × 10^6^ W/m^2^, and equivalently, *E*_0_ = 3.8 × 10^4^ V/m, since the relationship between intensity and the normalized electric ﬁeld is *I* = 0.5*n*𝜖c_0_*E*^2^ [*7*]. The distribution of the heat generation rate near the hotspot in GaN is shown in Fig. 2b in the main text, consistent with the hemi-ellipsoidal distribution of the electric ﬁeld. Notably, not all of the laser power has been converted to thermal energy. For example, the total heat generation for the laser power of 5.42 mW is 3.14 × 10^-4^ mW. The thermal energy generated within the optically induced hotspot is subsequently dissipated within the GaN substrate.

# S4. Details of first-principles calculations and phonon BTE calculations

In the phonon BTE given by Eq. (1) in the main text, *e_ω_*_,_*_p_*_,_**_s_** = *e*(**r**,**s**,*ω*,*p*) = *ћωD_p_*(*ω*)*n*(**r**,**s**,*ω*,*p*) donates the volumetric energy density of phonons at position **r** in direction **s** with frequency *ω*, polarization *p*, and wave vector **q**, where *n = n*(**r**,**s**,*ω*,*p*) is the phonon distribution function. The equivalent phonon temperature *T_ω_*_,_*_p_* can be obtained from the summation of energy density over phonons with frequency *ω* and polarization *p* according to [*8,9*], where *C_ω_*_,_*_p_* is the phonon heat capacity. The left-hand side of Eq. (1) is the phonon transport term, while the right-hand side is the scattering term that is dominated by phonon-phonon scattering, phonon-impurity scattering, and electron-phonon scattering in the bulk system under optical/electric excitations [*10*]. The phonon-phonon scattering and phonon-impurity scattering (only considering isotope scattering [*11*]) are usually described by the relaxation time approximation [*12*], and the electron-phonon scattering can be reduced to a mode-level energy transfer rate term [*13*]. This is the energy transferred from electrons to the phonon mode in unit volume and unit time at a certain equivalent electron temperature *T*_e_, as determined by the Fermi golden rule [*14*]:

(S1)

Here, (*n*, *m*) are the band indices; **k** is the wave vector of electrons with the distribution function *f*; *M* is the electron-phonon coupling matrix. The local electrons are assumed in the equilibrium Fermi-Dirac distribution with an equivalent temperature *T*_e_ in the first-principles calculations, which can be determined from the total energy power density applied by the incident Raman laser in experiments [*14*]. The Fröhlich coupling of electrons with LO phonons is rigorously addressed within the framework of our first-principles calculations. Note that it is the proportion of energy obtained by different phonon modes that matters, i.e., . The actual phonon mode-level heat generation rate is then determined by , where *Q* is the total heat generation rate distribution from electromagnetic simulations.

Phonon properties needed for solving the phonon BTE include the mode-level phonon group velocity *v_ω_*_,_*_p_*, heat capacity *C_ω_*_,_*_p_*, relaxation time *τ_ω_*_,_*_p_*, and heat generation rate . The *v_ω_*_,_*_p_*, *C_ω_*_,_*_p_*, and *τ_ω_*_,_*_p_* are calculated using harmonic and anharmonic lattice dynamics based on the QUANTUM-ESPRESSO [*15*] and ShengBTE [*16*] packages. For wurtzite GaN, a supercell of 4 × 4 × 4 and the fifth nearest atom neighbor is considered to obtain the third-order anharmonic interatomic force constant. We use 20 × 20 × 20 q-points to sample the Brillouin zone. The thermal conductivity (269 W/Mk at 300 K) calculated using the full iterative method agrees with well with those in the literature [*17*]. The mode-level heat generation rate is determined from the electron-phonon interaction calculations that are implemented in the electron-phonon Wannier (EPW) package [*18*]. The electron-phonon coupling matrix elements are firstly calculated on the coarse meshes and are then interpolated to 60 × 60 × 60 k-point and 40 × 40 × 40 q-point meshes to calculate the electron-phonon energy generation rate with our modified codes. In our calculations, the electrons satisfy the equilibrium distribution under *T*_e_ that is determined by the total power density of thermal generation, while phonons are maintained at the ambient temperature, *T*_ph_ = 300 K [*14*]. Fig. S7(a-c) shows the mode-level energy transfer rate from the electron system with equivalent temperature *T*_e_ = 900 K, 600 K, and 400 K to the phonon system that is maintained at 300 K in GaN. Different equivalent electron temperatures also represent different carrier concentrations that are the integral of the product of electron density of states and Fermi-Dirac distribution function with *T*_e_. It shows that the relative energy transfer rate among different phonons is roughly the same at different electron temperatures. As mentioned in the main text, it is the proportion of energy obtained by different phonon modes that matters. We quantitatively analyze three phonon groups as illustrated by the gray regions in Fig. S7(a). Group 1 includes all acoustic phonons. Groups 2 and 3 include phonons with frequencies ranging from 540 cm^-1^ to 570 cm^-1^ and from 710 cm^-1^ to 740 cm^-1^, which are associated with *E*_2_(TO) and *A*_1_(LO) phonon modes (not exactly the same), respectively. The proportion of energy obtained by these three phonon groups is illustrated in Fig. S7(d), which is shown to be nearly independent of the electron temperature/carrier concentration. Meanwhile, the proportion of energy obtained by these three phonon groups when adopting different k-point and q-point meshes are given in Fig. S8, which shows good numerical convergence. Additionally, Fig. S9 shows calculated e-ph scattering rates (average) in GaN at 300 K. For carriers in both conduction bands and valence bands, the scattering rate is very small within 0.1eV (≈ a LO phonon energy) of the band edges, indicating that scattering with LO phonons is the dominant process. The empirical Fröhlich scattering rate with LO phonons (red lines) is also computed as a comparison [*19*]. It shows good agreement with first-principle calculations near the band edges but clear discrepancy when far away from the band edges, especially for the valence bands. This arises from the fact that the larger DOS due to the presence of multiple valence bands causes more significant scattering from “nonpolar” phonon modes other than the LO phonons [*19*]. Overall, the strong LO polar scattering dominates near the band edges and contributes to increased scattering rates along with the nonpolar part when far away from the band edges.

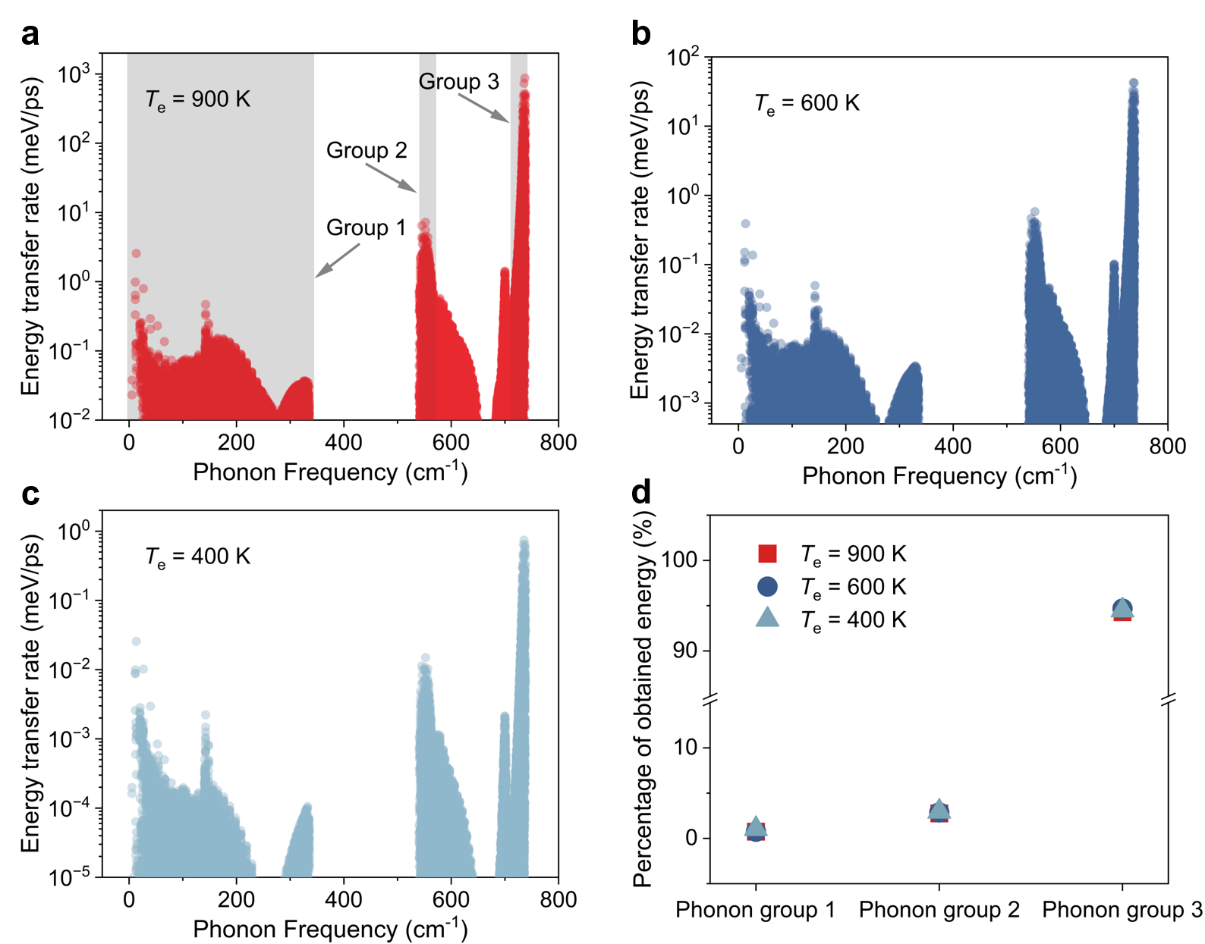


# Fig. S7

The mode-level energy transfer rate from the electron system with temperature *T*_e_ = (**a**) 900 K, (**b**) 600 K, (**c**) 400 K to the phonon system that is maintained at 300 K in GaN. (**d**) The proportion of energy obtained by three phonon groups as shown by the gray regions in (**a**).





# Fig. S8

The proportion of energy obtained by three phonon groups as shown by the gray regions in Fig. S7(**a**) calculated from first-principle calculations with 40 × 40 × 40, 50 × 50 × 50, 60 × 60 × 60 k-point and q-point meshes for *T*_e_ = 900K.





# Fig. S9

Calculated e-ph scattering rates in GaN at 300 K (the VBM is set as 0 eV). The symbols are e-ph scattering rates from first-principle calculations, and the red lines are the empirical Fröhlich scattering rates (*17*).

For calculating phonon properties of wurtzite AlN, a supercell of 5×5×3 and the fifth nearest atom neighbor is considered to obtain the third-order anharmonic interatomic force constant. We use 45×45×45 q-points to sample the Brillouin zone. The thermal conductivity (296 W/mK at 300 K) calculated using the full iterative method agrees well with those in the literature [*20*]. For zinc-blende BAs, the phonon properties are extracted from the FourPhonon packages [*21*]. The thermal conductivity is 2464 W/mK when only considering the three-phonon processes and is 1222 W/mK when considering both three-phonon and four-phonon processes at 300 K, which agrees well with those in the literature [*21*]. When performing the electron-phonon interaction calculations for both AlN and BAs in the EPW package, the electron-phonon coupling matrix elements are firstly calculated on the coarse meshes and are then interpolated to 40 × 40 × 40 k-point and 40 × 40 × 40 q-point meshes to calculate the electron-phonon energy generation rate. For illustrative purposes, the temperatures of electrons and phonons are maintained at 900 K and 300 K, respectively. The calculated phonon dispersion and phonon mode-level heat generation rate for AlN and BAs are illustrated in Fig. S12.

After obtaining mode-level phonon properties from first-principles calculations, the phonon BTE calculations are implemented in the GiftBTE package [*22*]. For predicting phonon temperature rises near the excited nanoscale hotspot under this tip-enhanced Raman measurement setup, a rectangular computational domain 20 × 20 × 10 μm^3^ is considered, which is large enough to mimic a bulk system. The top surface in contact with the nanotip is set as adiabatic, while the other five surfaces are thermalizing boundaries with a temperature of 300 K. The spatial heat generation rate distribution calculated from electromagnetic simulations is taken as the total heat generation rate in the phonon BTE calculations. The sequential iterative scheme with second-order spatial accuracy is applied. After the convergence test, 128 discretized phonon propagation directions, and 215378 mesh grids are employed.

For the case of hemispherical nanoscale hotspots in a simple 3D bulk system as shown in Fig. 3a in the main text, uniform heat generation within hotspots is applied. The value of uniform heat generation rate is arbitrary since it will not affect the ratio of the optical phonon temperature rise to the acoustic phonon temperature rise, i.e., the magnitude of phonon nonequilibrium. The size of the cubic computational domain is set to be large enough to be regarded as a bulk system (more than 10 times the phonon mean free path). The top surface is set as adiabatic, while the other surfaces are thermalizing boundaries with a temperature of *T*_0_. The sequential iterative scheme with second-order spatial accuracy is employed in the GiftBTE package. Two phonon bands, i.e., acoustic and optical phonon bands are applied to quantify the magnitude of phonon nonequilibrium. After the convergence test, 128 discretized phonon propagation directions, 25 phonon bands and about 200,000 mesh grids are employed for different hotspots in phonon BTE calculations.

# S5. Phonon temperature distributions

Using the first-principles based phonon BTE, we have calculated the phonon temperature distributions in the GaN substrate in the tip-enhanced Raman measurements. Fig. S10 and Fig. S11 show the top view (*z*=0 plane) and cross section view (*y*=0 plane) of the phonon temperature distributions, respectively. The *A*_1_(LO) phonon temperature, *E*_2_(TO) phonon temperature, average temperature of acoustic phonons, and also the Fourier’s law predictions are shown. The BTE calculated results shown in Fig. 2c in the main text are Raman intensity weighted for the temperature distributions over the space domain as shown in Fig. S10 [*23*]. Compared the temperature distribution from the Fourier’s law, evident localized high temperature rises are observed especially for optical phonons. As we analyzed in the main text, this is caused by the strong Fröhlich coupling of electrons with LO phonons and the large acoustic-optical frequency gap.


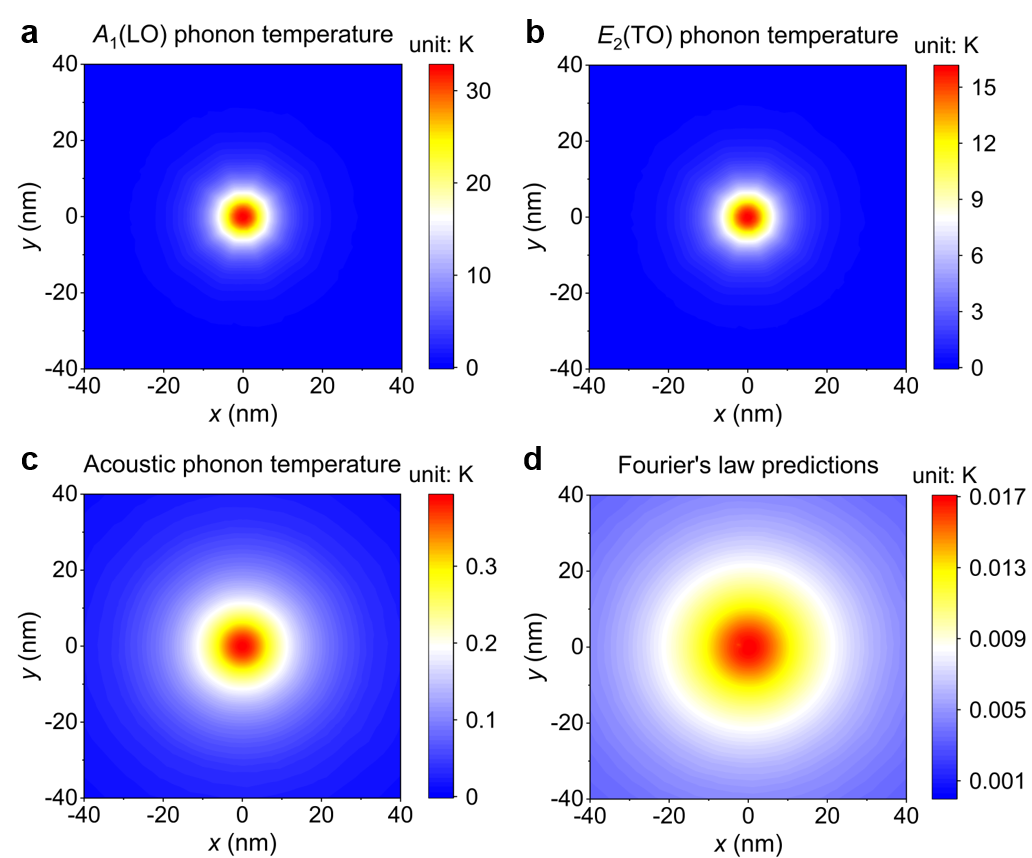


# Fig. S10

Top view of the phonon temperature distribution in the GaN substrate (*z*=0 plane) for (**a**) *A*_1_(LO) phonon mode (**b**) *E*_2_(TO) phonon mode (**c**) acoustic phonons calculated from the first-principles based phonon BTE and (**d**) Fourier’s law predictions.


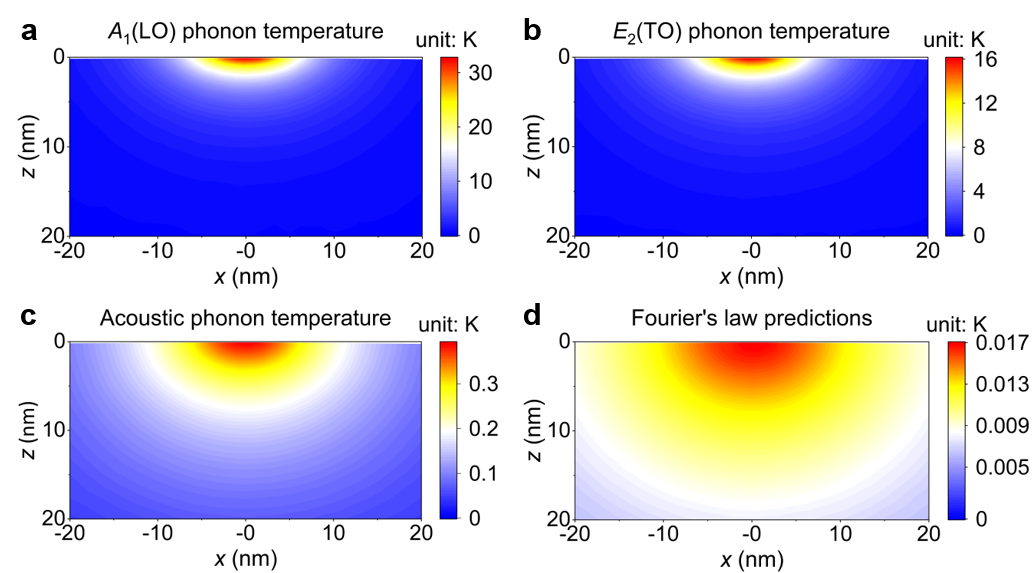


# Fig. S11

Cross section view of the phonon temperature distribution within the GaN substrate (*y*=0 plane) for (**a**) *A*_1_(LO) phonon mode (**b**) *E*_2_(TO) phonon mode (**c**) acoustic phonons calculated from the first-principles based phonon BTE and (**d**) the Fourier’s law predictions.

# S6. Phonon dispersion and mode-level heat generation rate for AlN and BAs

The phonon dispersion and model-level heat generation rate for AlN and BAs obtained from first-principle calculations are presented in Fig. S12. A peak of heat generation rate for LO phonons near the Brillouin zone center is shown for AlN. This is consistent with our analysis that the large electronegativity difference between the aluminum atom and the nitride atom leads to strong Fröhlich coupling and highly selective phonon excitation. It is worth noting that some other optical phonons and acoustic phonons also have relatively large heat generation rates, similar to results found in the literature [*24*]. For BAs, the heat generation rate is relatively uniformly distributed across different phonon modes. However, the zone-center optical phonons still occupy a large portion of the energy transferred from electrons, as consistent with results in [*25*]. A significant AO gap is obtained for BAs due to the large mass difference between the boron atom and the arsenide atom, which is consistent with previous studies [*26*].


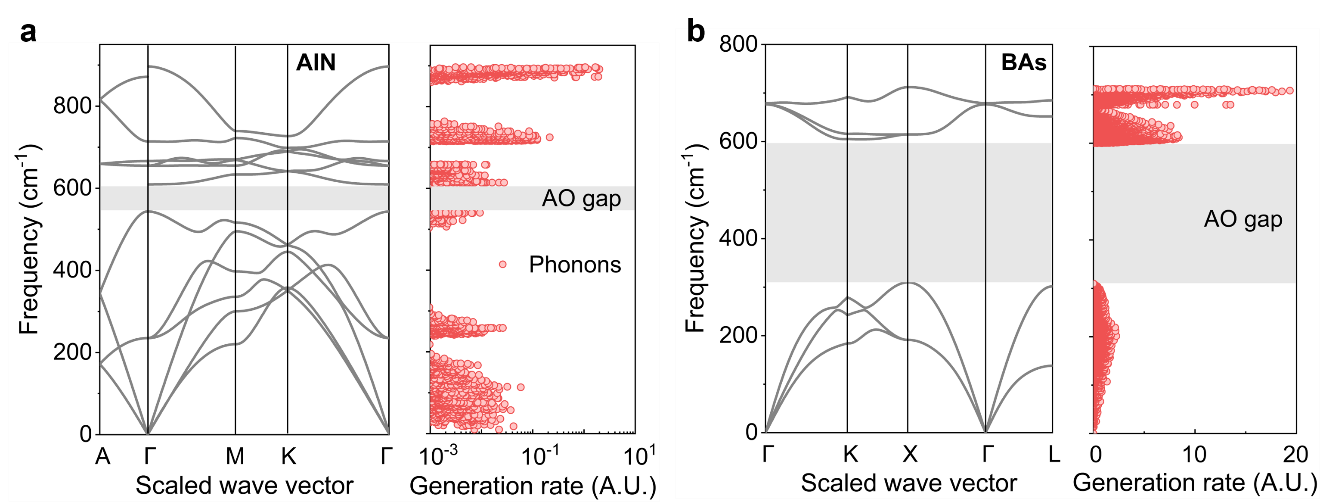


# Fig. S12

The calculated phonon dispersion and phonon mode-level heat generation rate for (**a**) AlN and (**b**) BAs. Each red symbol corresponds to a certain phonon mode. The gray regions represent the AO frequency gap.

# References

(1) Harima, H. Properties of GaN and related compounds studied by means of Raman scattering. *Journal of Physics: Condensed Matter* **2002**, *14*, R967.

(2) Song, D. Y.; Nikishin, S. A.; Holtz, M.; Soukhoveev, V.; Usikov, A.; Dmitriev, V. Decay of zone-center phonons in GaN with A1, E1, and E2 symmetries. *Journal of Applied Physics* **2007**, *101*, 053535.

(3) Link, A.; Bitzer, K.; Limmer, W.; Sauer, R.; Kirchner, C.; Schwegler, V.; Kamp, M.; Ebling, D.; Benz, K. Temperature dependence of the E_2_ and A_1_ (LO) phonons in GaN and AlN. *Journal of Applied Physics* **1999**, *86*, 6256-6260.

(4) Sodan, V.; Kosemura, D.; Stoffels, S.; Oprins, H.; Baelmans, M.; Decoutere, S.; Wolf, I. D. Experimental benchmarking of electrical methods and μ-Raman spectroscopy for channel temperature detection in AlGaN/GaN HEMTs. *IEEE Transactions on Electron Devices* **2016**, *63*, 2321-2327.

(5) Losurdo, M.; Giangregorio, M. M.; Bianco, G. V.; Suvorova, A. A.; Kong, C.; Rubanov, S.; Capezzuto, P.; Humlicek, J.; Bruno, G. Size dependence of the dielectric function of silicon-supported plasmonic gold nanoparticles. *Physical Review B* **2010**, *82*, 155451.

(6) Hao, J.; Zhou, L.; Qiu, M. Nearly total absorption of light and heat generation by plasmonic metamaterials. *Physical Review B* **2011**, *83*, 165107.

(7) Loudon, R. The propagation of electromagnetic energy through an absorbing dielectric. *Journal of Physics A: General Physics* **1970**, *3*, 233.

(8) Hu, Y.; Feng, T.; Gu, X.; Fan, Z.; Wang, X.; Lundstrom, M.; Shrestha, S. S.; Bao, H. Unification of nonequilibrium molecular dynamics and the mode-resolved phonon Boltzmann equation for thermal transport simulations. *Physical Review B* **2020**, *101* 155308.

(9) Xu, J.; Hu, Y.; Ruan, X.; Wang, X.; Feng, T.; Bao, H. Nonequilibrium phonon transport induced by finite sizes: effect of phonon-phonon coupling. *Physical Review B* **2021**, *104*, 104310.

(10) Bao, H.; Chen, J.; Gu, X.; Cao, B. A Review of simulation methods in micro/nanoscale heat conduction. *ES Energy & Environment* **2018**, *1*, 16-55.

(11) Berglund, M.; Wieser, M. E. Isotopic compositions of the elements 2009 (IUPAC Technical Report). *Pure and Applied Chemistry* **2011**, *83*, 397-410.

(12) Majumdar, A. Microscale heat conduction in dielectric thin films. *Journal of Heat Transfer* **1993**, *115*, 7-16.

(13) Pop, E.; Sinha, S.; Goodson, K. E. Heat generation and transport in nanometer-scale transistors. *Proceedings of the IEEE* **2006**, *94*, 1587-1601.

(14) Minamitani, E. Ab initio analysis for the initial process of Joule heating in semiconductors. *Physical Review B* **2021**, *104*, 085202.

(15) Giannozzi, P.; Baroni, S.; Bonini, N.; Calandra, M.; Car, R.; Cavazzoni, C.; Ceresoli, D.; Chiarotti, G. L.; Cococcioni, M.; Dabo, I.; et al. QUANTUM ESPRESSO: a modular and open-source software project for quantum simulations of materials. *Journal of Physics: Condensed Matter* **2009**, *21*, 395502.

(16) Li, W.; Carrete, J.; A. Katcho, N.; Mingo, N. ShengBTE: A solver of the Boltzmann transport equation for phonons. *Computer Physics Communications* **2014**, *185*, 1747-1758.

(17) Ma, J.; Li, W.; Luo, X. Intrinsic thermal conductivities and size effect of alloys of wurtzite AlN, GaN, and InN from first-principles. *Journal of Applied Physics* **2016**, *119*, 125702.

(18) Poncé, S.; Margine, E. R.; Verdi, C.; Giustino, F. EPW: Electron–phonon coupling, transport and superconducting properties using maximally localized Wannier functions. *Computer Physics Communications* **2016**, *209*, 116-133.

(19) Jhalani, V. A.; Zhou, J.-J.; Bernardi, M. Ultrafast hotcarrier dynamics in GaN and its impact on the efficiency droop. *Nano Letters* **2017**, *17*, 5012-5019.

(20) Li, W.; Mingo, N. Thermal conductivity of bulk and nanowire InAs, AlN, and BeO polymorphs from first principles. *Journal of Applied Physics* **2013**, *114*, 125702.

(21) Han, Z.; Yang, X.; Li, W.; Feng, T.; Ruan, X. FourPhonon: An extension module to ShengBTE for computing four-phonon scattering rates and thermal conductivity. *Computer Physics Communications* **2022**, *270*, 108179.

(22) Hu, Y.; Jia, R.; Xu, J.; Sheng, Y.; Wen, M.; Lin, J.; Shen, Y.; Bao, H. GiftBTE: an efficient deterministic solver for non-gray phonon Boltzmann transport equation. *Journal of Physics: Condensed Matter* **2024**, *36*, 025901.

(23) Wang, R.; Zobeiri, H.; Xie, Y.; Wang, X.; Zhang, X.; Yue, Y. Distinguishing optical and acoustic phonon temperatures and their energy coupling factor under photon excitation in nm 2D materials. *Advanced Science* **2020**, *7*, 2000097.

(24) Quan, Y.; Chen, Y.; Liao, B. Significant phonon drag effect in wide band gap GaN and AlN. *Physical Review B* **2023**, *107*, 245202.

(25) Sadasivam, S.; Chan, M. K. Y.; Darancet, P. Theory of thermal relaxation of electrons in semiconductors. *Physical Review Letters* **2017**, *119*, 136602.

(26) Lindsay, L.; Broido, D. A.; Reinecke, T. L. First-principles determination of ultrahigh thermal conductivity of boron srsenide: a competitor for diamond? *Physical Review Letters* **2013**, *111*, 025901.
